# Supplementary material for: Timing matters in the use of renin-angiotensin system modulators and COVID-related cognitive and cerebrovascular dysfunction
Source: PLoS One. 2024 Jul 29;19(7):e0304135. doi: 10.1371/journal.pone.0304135 (PMC11285960; doi:10.1371/journal.pone.0304135)
Supplement: S1 Raw images — (PDF) [file pone.0304135.s001.pdf]

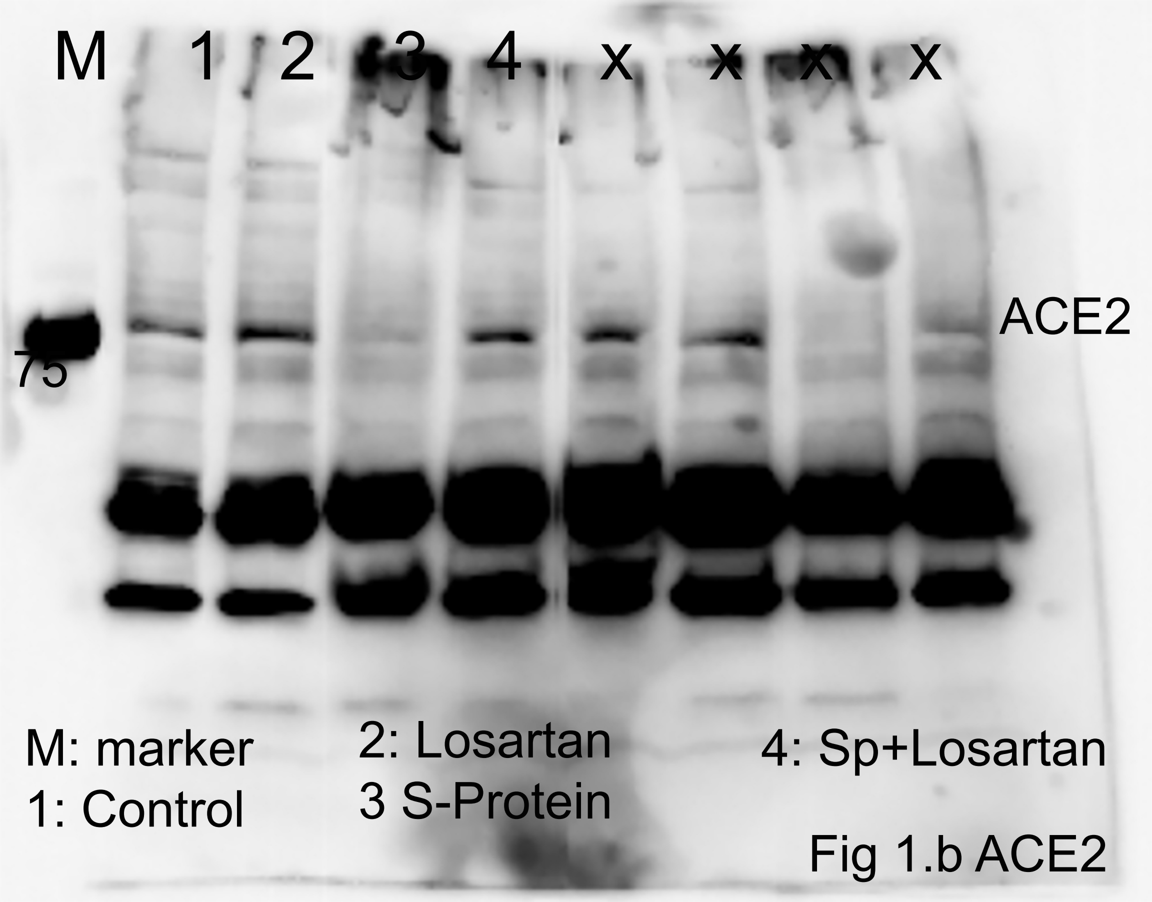
M 1 2 3 4 x x x x
75
ACE2
M: marker
1: Control
2: Losartan
3 S-Protein
4: Sp+Losartan

Fig 1.b ACE2

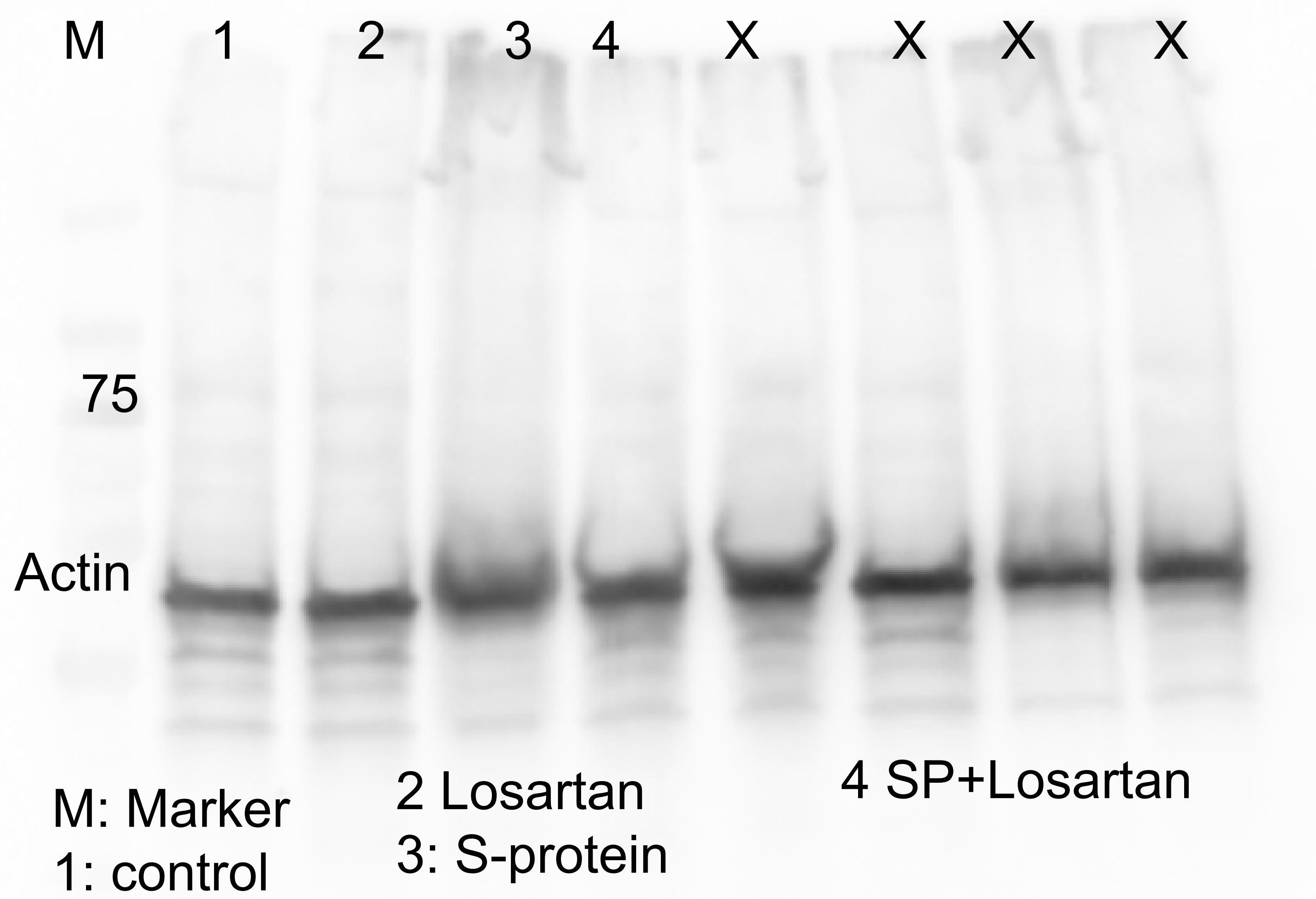
M
1
2
3
4
X
X
X
X
75
Actin
M: Marker
1: control
2 Losartan
3: S-protein
4 SP+Losartan

Fig 1. b actin

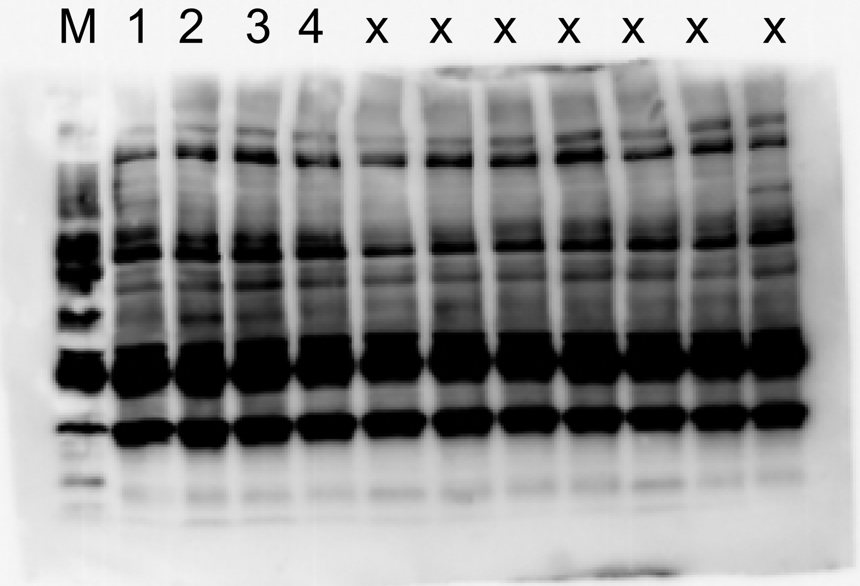

M: Marker
1: Control
2 S-Protein

3: Losartan+Sp
4: SP+Losartan

Fig 2.b Cl. Cas-3

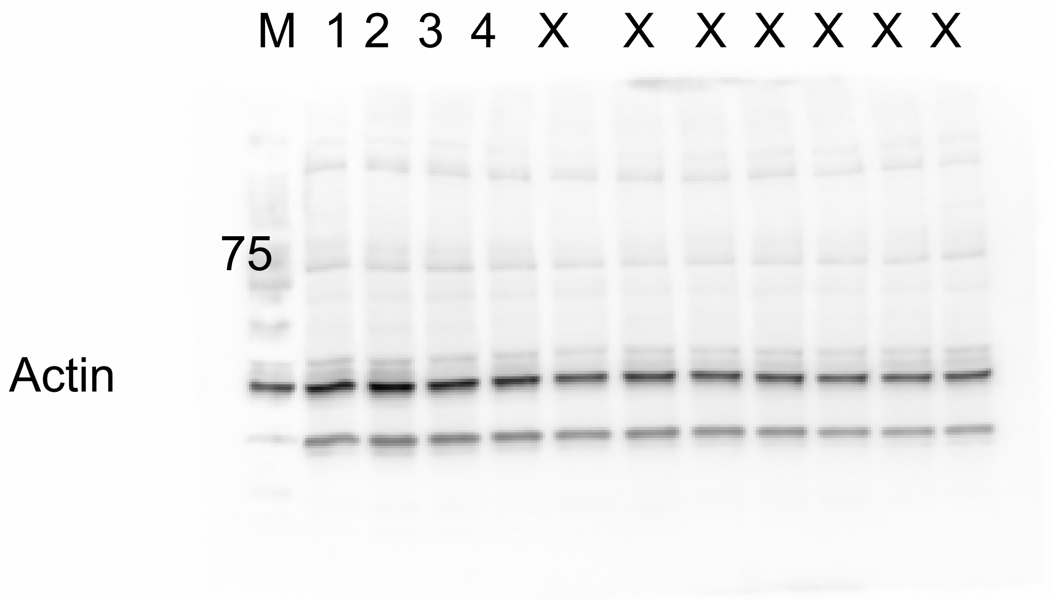

M: marker
1: Control
2S-Protein
3 Losartan+SP
4 SP+Losartan

Fig 2.b Actin
