# Supplementary material for: Timing matters in the use of renin-angiotensin system modulators and COVID-related cognitive and cerebrovascular dysfunction
Source: PLoS One. 2024 Jul 29;19(7):e0304135. doi: 10.1371/journal.pone.0304135 (PMC11285960; doi:10.1371/journal.pone.0304135)
Supplement: Attachment — Submitted filename: Response to Reviewers.docx [file pone.0304135.s002.docx]

**Response to Journal Requirements:**

1. Please ensure that your manuscript meets PLOS ONE's style requirements, including those for file naming. The PLOS ONE style templates can be found at

[https://journals.plos.org/plosone/s/file?id=wjVg/PLOSOne_formatting_sample_main_body.pdf](https://urldefense.com/v3/__https:/journals.plos.org/plosone/s/file?id=wjVg*PLOSOne_formatting_sample_main_body.pdf__;Lw!!I6mtC9TwIjWPYSMa4w!17r_JUp4TYoDZS_z6QmbugBJzzdb7piqaImZ--nU1rA4Uvwnv6la4RfhptYIJ__IVvny9anjSKv9s3CDPtNaHuV1$) and 
[https://journals.plos.org/plosone/s/file?id=ba62/PLOSOne_formatting_sample_title_authors_affiliations.pdf](https://urldefense.com/v3/__https:/journals.plos.org/plosone/s/file?id=ba62*PLOSOne_formatting_sample_title_authors_affiliations.pdf__;Lw!!I6mtC9TwIjWPYSMa4w!17r_JUp4TYoDZS_z6QmbugBJzzdb7piqaImZ--nU1rA4Uvwnv6la4RfhptYIJ__IVvny9anjSKv9s3CDPoKLlDXX$)

The file naming was adjusted to follow PLOS ONE’s style requirements

2. To comply with PLOS ONE submissions requirements, in your Methods section, please provide additional information regarding the experiments involving animals and ensure you have included details on (1) methods of sacrifice, and (2) efforts to alleviate suffering.

We have updated the method section as directed with the following: “Animals were sacrificed using carbon dioxide and cervical dislocation. Buprenorphine 0.1 mg/kg body weight was injected subcutaneously upon detecting any signs of animal distress.”

3. We note that the grant information you provided in the ‘Funding Information’ and ‘Financial Disclosure’ sections do not match.

When you resubmit, please ensure that you provide the correct grant numbers for the awards you received for your study in the ‘Funding Information’ section.

We have updated the funding information.

4. Thank you for stating the following in the Competing Interests section:

"Some data were presented as an abstract at the International Stroke Conference 2023 and the MIDYEAR 2023 Clinical Meeting & Exhibition.
The authors have declared that no competing interests exist.
Funding:This study was supported by American Heart Association 23AIREA1045073 to MA."

Please confirm that this does not alter your adherence to all PLOS ONE policies on sharing data and materials, by including the following statement: "This does not alter our adherence to  PLOS ONE policies on sharing data and materials.” (as detailed online in our guide for authors [http://journals.plos.org/plosone/s/competing-interests).](https://urldefense.com/v3/__http:/journals.plos.org/plosone/s/competing-interests).__;!!I6mtC9TwIjWPYSMa4w!17r_JUp4TYoDZS_z6QmbugBJzzdb7piqaImZ--nU1rA4Uvwnv6la4RfhptYIJ__IVvny9anjSKv9s3CDPj9namIj$)

If there are restrictions on sharing of data and/or materials, please state these. Please note that we cannot proceed with consideration of your article until this information has been declared.

Please include your updated Competing Interests statement in your cover letter; we will change the online submission form on your behalf.

“This does not alter our adherence to  PLOS ONE policies on sharing data and materials.” was added to the manuscript and the cover letter as directed

5. In the online submission form you indicate that your data is not available for proprietary reasons and have provided a contact point for accessing this data. Please note that your current contact point is a co-author on this manuscript. According to our Data Policy, the contact point must not be an author on the manuscript and must be an institutional contact, ideally not an individual. Please revise your data statement to a non-author institutional point of contact, such as a data access or ethics committee, and send this to us via return email. Please also include contact information for the third party organization, and please include the full citation of where the data can be found.

All relevant data are within the manuscript and its Supporting Information files. The online submission was updated accordingly

6. PLOS ONE now requires that authors provide the original uncropped and unadjusted images underlying all blot or gel results reported in a submission’s figures or Supporting Information files. This policy and the journal’s other requirements for blot/gel reporting and figure preparation are described in detail at [https://journals.plos.org/plosone/s/figures#loc-blot-and-gel-reporting-requirements](https://urldefense.com/v3/__https:/journals.plos.org/plosone/s/figures*loc-blot-and-gel-reporting-requirements__;Iw!!I6mtC9TwIjWPYSMa4w!17r_JUp4TYoDZS_z6QmbugBJzzdb7piqaImZ--nU1rA4Uvwnv6la4RfhptYIJ__IVvny9anjSKv9s3CDPgRDsnvl$) and [https://journals.plos.org/plosone/s/figures#loc-preparing-figures-from-image-files](https://urldefense.com/v3/__https:/journals.plos.org/plosone/s/figures*loc-preparing-figures-from-image-files__;Iw!!I6mtC9TwIjWPYSMa4w!17r_JUp4TYoDZS_z6QmbugBJzzdb7piqaImZ--nU1rA4Uvwnv6la4RfhptYIJ__IVvny9anjSKv9s3CDPhs4U9Yc$). When you submit your revised manuscript, please ensure that your figures adhere fully to these guidelines and provide the original underlying images for all blot or gel data reported in your submission. See the following link for instructions on providing the original image data: [https://journals.plos.org/plosone/s/figures#loc-original-images-for-blots-and-gels.](https://urldefense.com/v3/__https:/journals.plos.org/plosone/s/figures*loc-original-images-for-blots-and-gels.__;Iw!!I6mtC9TwIjWPYSMa4w!17r_JUp4TYoDZS_z6QmbugBJzzdb7piqaImZ--nU1rA4Uvwnv6la4RfhptYIJ__IVvny9anjSKv9s3CDPjVE46w1$) 
 In your cover letter, please note whether your blot/gel image data are in Supporting Information or posted at a public data repository, provide the repository URL if relevant, and provide specific details as to which raw blot/gel images, if any, are not available. Email us at plosone@plos.org if you have any questions.

The original uncropped images are submitted.

7. We note that you have included the phrase “data not shown” in your manuscript. Unfortunately, this does not meet our data sharing requirements. PLOS does not permit references to inaccessible data. We require that authors provide all relevant data within the paper, Supporting Information files, or in an acceptable, public repository. Please add a citation to support this phrase or upload the data that corresponds with these findings to a stable repository (such as Figshare or Dryad) and provide and URLs, DOIs, or accession numbers that may be used to access these data. Or, if the data are not a core part of the research being presented in your study, we ask that you remove the phrase that refers to these data.

The phrase was removed.

**Response to Reviewers**

Reviewer #1:

1. The authors must describe a technically sound piece of scientific research with data that supports the conclusions. Experiments must have been conducted rigorously, with appropriate controls, replication, and sample sizes

We thank the reviewer for their valuable comment. We assure the reviewer that we have adopted the NIH rigor policy and that we comply with transparent standards in our research. All our experiments have been rigorously designed. Our method section describes all experimental procedures in detail for transparency and reproducibility. Proper controls and animal randomization have been used to reduce bias. Our sample size was selected to ensure a power analysis at a significance level of P<0.05. Our animal studies used male and female h ACE-2 mice to avoid biological variables. Finally, all our antibodies and chemical reagent sources have been stated in our manuscript. These reagents have been authenticated by their vendor.

2. The figures lack legends that describe the statistical analyzes and sample sizes of each of the experimental groups.

We apologize for this misunderstanding. According to the PLOS One manuscript author guide, all the figure legends are included in the manuscript body and they include sample size and statistical method, as directed.

3. The authors must validate that the preclinical model used emulates the recruited patients. In the preclinical model, it is not specified whether they used only males or both males and females. The preclinical model used by the authors. Validation of the preclinical model is missing.

We apologize for the mistake and totally agree with the reviewer that the pre-clinical model must be validated. We and others have used hACE-2 mice in COVID-19 research. We have included the reference for studies using the animal model in our discussion. Both male and female hACE-2 mice have been used in our experimental design to avoid biological variables. We have updated our method section accordingly.

(1) Munoz-Fontela C, Dowling WE, Funnell SGP, Gsell PS, Riveros-Balta AX, Albrecht RA, et al. Animal models for COVID-19. Nature. 2020;586(7830):509-15.

(2) Burnett FN, Coucha M, Bolduc DR, Hermanns VC, Heath SP, Abdelghani M, et al. SARS-CoV-2 Spike Protein Intensifies Cerebrovascular Complications in Diabetic hACE2 Mice through RAAS and TLR Signaling Activation. Int J Mol Sci. 2023;24(22).

4. The patients received ACEI/ARB, however the mice received only ARB, losartan. The treatment with ACEi remains to be incorporated in the preclinical model.

We thank the reviewer for their constructive feedback. While patients received ACEI or ARB therapy, we exclusively used ARB, Losartan, in the preclinical study. This choice was based on our recent research demonstrating Losartan's efficacy in restoring RAS balance and mitigating spike protein-induced cerebrovascular dysfunction (2). We acknowledge that including ACE inhibitor therapy in our preclinical model would offer a more comprehensive reflection of the clinical arm. However, incorporating ACE inhibitors would require a complete repetition of the whole preclinical study, which is unfeasible. The following statement was included in the manuscript to reflect this limitation.

“Future studies should address the limitation of our preclinical model, which exclusively utilized ARB therapy, by incorporating ACEI to mimic the clinical arm where patients received either ARB or ACEI”

Reviewer #2: The paper is well written and addresses an important question of the timing of RAS modulators in COVID-19 related cognitive dysfunction. They show statistically better outcomes in patients receiving the medication post infection. I think the paper is acceptable in its current form.

Your feedback is greatly appreciated, and we're grateful for your time.

Reviewer #3: In this clinical/preclinical paper, the authors make an attempt to correlate RAS-inhibition before or post onset of Covid-19 with neurological dysfunction. While the preclinical part provides interesting results, appears to be well done and methodologically sound, I have major concerns with respect to the clinical part:

In general, the observed increase in neurological dysfunction in patients on RAS inhibition may be simply due to the fact that these patients were more severely ill, had higher BP levels to start with or cardio-renal problems etc. which afforded additional treatment with RAS inhibitors.

- The number of patients is relatively small. Meaningful statistics are difficult with such small numbers. - The proportion of black patients is too high to be representative for the US population, let alone the rest of the world.

- Data on comorbidities are lacking.

– No information on BP control

- No information on the methods to evaluate mental status - Coincidence of neurological problems: The number (91) with neurological dysfunction in RAS inhibitor- treated patients does not distinguish between i) one dysfunction in one patient or ii) several neurological problems in one patient.

We would like to extend our sincere gratitude for the time and effort the reviewer dedicated to reviewing our manuscript. The reviewer’s feedback has been instrumental in strengthening our manuscript. Below is our response to the reviewer’s comments

Firstly, we acknowledge that the clinical part of the study is a retrospective chart review, which inherently relies on electronic medical record documentation of subjective complaints. While we tried to identify objective measures of neurological dysfunction, such as CT scans or MRIs, not all patients underwent such evaluations due to various clinical reasons.

In response to your concern regarding the potential influence of patients' overall clinical condition, including severity of illness, baseline blood pressure levels, or cardiorenal problems, we have implemented several measures to address this issue. Specifically, we have incorporated additional data on comorbidities and blood pressure control into our analysis. These findings indicate that the two groups exhibit similarities in these parameters, thereby indicating that the observed disparities in neurological dysfunction are less likely to be solely attributable to baseline clinical characteristics.

We also acknowledge the high proportion of African American patients included in our study. As residents of the Southeast, where approximately half of the population is African American, it is not surprising that a significant number of African American patients were evaluated in our retrospective chart review. We appreciate your understanding of this demographic representation and assure you that it does not impact the validity of our findings.

Minor problems:

- line 318: Ang II has the same affinity to AT1R and AT2R. The difference comes rather from the number of receptors in a given tissue under given circumstances.

We apologize for this mistake, and it was corrected in the manuscript to the following “Ang II binds with two main receptors, AT­_1_R and AT_2_R, and triggers different biological responses.”

- line 320: The AT2R-axis of the "protective RAS" needs to be referenced, e.g. Steckelings et al. Pharm. Reviews 2022.

The review is being referenced in the manuscript.

- line 323: The protective RAS is not only constituted by the Ang 1-7/Mas axis but also via the AT2R. Stimulation of the unopposed AT2R could be a mechanism of the protective effects of Losartan post SP in the animals.

The following challenges were made to enhance the clarity of the manuscript:

However, AT_2_R activation contributes to more vascular protective effects such as vasodilation and anti-inflammatory effects (3). Moreover, ACE-2 is an enzyme that catalyzes the degradation of Ang II to Ang-(1-7), and the resultant molecules activate the Mas receptor which results in protective actions (4-6). Therefore, Ang-(1-7)/Mas axis and the AT_2_R constitute the protective arm of the RAS.

Additionally, unopposed AT_2_R stimulation could be a mechanism underlying the protective effect of Losartan demonstrated in the study.

- line 348: Meta-analysis on Covid-19/ RAS inhibition by Gnanenthiran SR et al., J Am Heart Assoc. 2022 should be included.

The Meta-analysis was included in the manuscript.

In 2022, Gnanenthiran et al. published a meta-analysis to assess the safety and efficacy of RAS inhibiton in adults with COVID‐19. The authors found no significant difference in all-cause mortality between patients receiving RAS inhibition = therapy and those without it. However, there was a borderline decrease in acute myocardial infarction among patients on RASi. On the other hand, there was an increased risk of acute kidney injury (AKI) associated with RASi use, particularly in hospitalized patients. Despite this increased risk of AKI, there was no increase in the need for dialysis or other adverse outcomes such as congestive cardiac failure, stroke, or venous thromboembolism (7).

1.	Munoz-Fontela C, Dowling WE, Funnell SGP, Gsell PS, Riveros-Balta AX, Albrecht RA, et al. Animal models for COVID-19. Nature. 2020;586(7830):509-15.

2.	Burnett FN, Coucha M, Bolduc DR, Hermanns VC, Heath SP, Abdelghani M, et al. SARS-CoV-2 Spike Protein Intensifies Cerebrovascular Complications in Diabetic hACE2 Mice through RAAS and TLR Signaling Activation. Int J Mol Sci. 2023;24(22).

3.	Steckelings UM, Widdop RE, Sturrock ED, Lubbe L, Hussain T, Kaschina E, et al. The Angiotensin AT(2) Receptor: From a Binding Site to a Novel Therapeutic Target. Pharmacol Rev. 2022;74(4):1051-135.

4.	Raffai G, Durand MJ, Lombard JH. Acute and chronic angiotensin-(1-7) restores vasodilation and reduces oxidative stress in mesenteric arteries of salt-fed rats. Am J Physiol Heart Circ Physiol. 2011;301(4):H1341-52.

5.	Santos RA, Campagnole-Santos MJ, Andrade SP. Angiotensin-(1-7): an update. Regul Pept. 2000;91(1-3):45-62.

6.	Khajehpour S, Aghazadeh-Habashi A. Targeting the Protective Arm of the Renin-Angiotensin System: Focused on Angiotensin-(1-7). J Pharmacol Exp Ther. 2021;377(1):64-74.

7.	Gnanenthiran SR, Borghi C, Burger D, Caramelli B, Charchar F, Chirinos JA, et al. Renin-Angiotensin System Inhibitors in Patients With COVID-19: A Meta-Analysis of Randomized Controlled Trials Led by the International Society of Hypertension. J Am Heart Assoc. 2022;11(17):e026143.
