# Supplementary material for: Timing matters in the use of renin-angiotensin system modulators and COVID-related cognitive and cerebrovascular dysfunction
Source: PLoS One. 2024 Jul 29;19(7):e0304135. doi: 10.1371/journal.pone.0304135 (PMC11285960; doi:10.1371/journal.pone.0304135)
Supplement: Attachment — Submitted filename: Response to Reviewers.docx [file pone.0304135.s003.docx]

Dear Editor,

The authors would like to thank all the reviewers (1, 2, and 3) for their invaluable feedback and constructive criticism of our manuscript titled "Timing Matters in the Use of Renin-Angiotensin System Modulators and COVID-Related Cognitive and Cerebrovascular Dysfunction." Their time and expertise are greatly appreciated.

I am writing in response to Reviewer #3's feedback on our study. The authors appreciate Reviewer #3's time and effort in providing a thorough evaluation of our study.

We understand Reviewer #3's concerns regarding the clinical part of our study and the potential limitations associated with it. However, we respectfully disagree with the suggestion to delete the clinical part of the study. We believe that the clinical data significantly contribute to the overall findings and provide valuable insights into the correlation between RAAS inhibition and neurological dysfunction in patients with COVID-19. The clinical data strengthens the scientific merit of our research.

Here is a detailed response to Reviewer #3's concerns regarding the small sample size, the high proportion of African Americans, and the lack of rigorous testing in all patients.

Small sample size: We understand the concerns raised by the reviewer regarding the sample size. However, we would like to emphasize that the goal of this study was to shed light on the potential correlation between RAAS inhibition and neurological dysfunction following COVID-19 exposure. In this study, we tried to identify areas for further investigation. While we acknowledge the importance of having a sufficient number of patients in the clinical study, the findings that we observed based on the **295 patients** included in the study can serve as a foundation for future research projects with a larger sample of patients.

Lack of rigorous testing in all patients: Firstly, we acknowledge that the clinical part of the study is a retrospective chart review, which inherently relies on electronic medical record documentation of subjective complaints. We acknowledge the concern raised regarding the absence of comprehensive neurological evaluations, such as CT scans or MRIs, in all patients within our retrospective chart review. It is important to note that the decision for these objective imaging tests was subject to the discretion of attending physicians based on clinical indications and resource availability. Given the retrospective nature of our study and the diversity of patient presentations, not all individuals underwent the same diagnostic procedures. While this may introduce variability in the data collection process, it does not compromise the integrity of our findings. We analyzed the available clinical data to identify trends and associations, ensuring transparency in our reporting of both objective and subjective measures of neurological dysfunction. Furthermore, our study's primary focus was on exploring potential correlations between RAAS inhibition and neurological outcomes in the context of COVID-19, rather than exclusively relying on imaging findings. We have acknowledged this limitation in our revised manuscript and provided

interpretations of our results within the framework of available clinical data. Despite the variability in diagnostic evaluations, our study still provides valuable insights into the relationship between RAAS modulation and neurological dysfunction post-COVID-19 exposure, warranting further investigation.

High proportion of African Americans: We acknowledge the high proportion of African American patients included in our study. The data was collected from St. Joseph’s/Candler Health System located in Savannah, Georgia. “*In 2021, there were 1.41 times more Black or African American (non-Hispanic) residents (76k people) in Savannah, GA, than any other race or ethnicity. There were 54k White (non-Hispanic) and 4.01k White (Hispanic) residents, the second and third most common ethnic groups* [1]”. Therefore, it is not surprising that a significant number of African American patients were evaluated in our retrospective chart review. We appreciate your understanding of this demographic representation and assure you that it does not impact the validity of our findings. To provide further clarity, we have included a table detailing the distribution of African American and White patients included in our study. As shown in the table, the majority of the patients were White. While we acknowledge the high proportion of African American patients included in our study; we believe that this demographic diversity enriches our findings and underscores the importance of considering racial disparities in healthcare outcomes.

[1] <https://datausa.io/profile/geo/savannah-ga#race_and_ethnicity>

| Characteristic | ACEI/ARB prior to admission (n=177) | No ACEI/ARB prior to admission (n=118) |
| --- | --- | --- |
| Caucasian | 97 (55%) | 68 (58%) |
| African American | 78 (44% | 44 (37%) |
| Other | 2 (1%) | 6 (5%) |

We have revised the manuscript to clarify those issues, highlight any limitations within the study, and underscore the need for further studies to validate the findings. We believe that those changes will strengthen the quality and impact of our manuscript.

We respectfully request that the journal reconsider its decision to remove the clinical part of our study.

Thank you for considering our request.

Sincerely,

**Maha Coucha, B.Pharm., Ph.D**

Associate Professor

Department of Pharmaceutical Sciences

School of Pharmacy

**Office**: 912.201.8146

Fax: 912.201.8153

**Email:** [mcouch@southuniversity.edu](mailto:mcouch@southuniversity.edu)

709 Mall Boulevard

Savannah, GA 31406

[**southuniversity.ed**](http://southuniversity.edu/)
